# Genetic variability in *Neisseria meningitidis* strains isolated in a Japanese hospital

**DOI:** 10.1016/j.ijregi.2024.100511

**Published:** 2024-12-05

**Authors:** Kazuyoshi Gotoh, Shinnosuke Fukushima, Hideharu Hagiya, Shuma Tsuji, Koji Iio, Osamu Matsushita

**Affiliations:** 1 Department of Medical Laboratory Science, Okayama University Graduate School of Health Sciences, Okayama, Japan; 2 Department of Bacteriology, Okayama University Graduate School of Medicine, Dentistry and Pharmaceutical Sciences, Okayama, Japan; 3 Department of Infectious Diseases, Okayama University Hospital, Okayama, Japan; 4 Microbiology Division, Clinical Laboratory, Okayama University Hospital, Okayama, Japan

**Keywords:** Antimicrobial resistance, Invasive meningococcal disease, Drug-resistant gene, Genome sequence

## Abstract

**Objectives:**

*Neisseria meningitidis* is a significant pathogen causing invasive meningococcal disease, posing clinical and public health concerns worldwide. This study aimed to investigate the genetic characteristics of *N. meningitidis* clinical isolates at Okayama University Hospital in Japan.

**Methods:**

Between 2018 and 2023, five clinical strains were isolated, of which three were subjected to the antimicrobial susceptibility testing and whole genetic analysis using MiSeq platform (Illumina, San Diego, CA, USA).

**Results:**

One non-groupable isolate, belonging to sequence types (STs)-11026 (ST-32 complex), exhibited non-susceptibility to penicillin G, with a five-mutation pattern (F504L, A510V, I515V, H541N, and I566V) in the *penA* amino acid sequence and additional mutations (XXXIV and N513Y) characteristic of a mosaic *penA* gene. The other two isolates, ST-1655 (ST-23 complex) with serogroup Y and ST-2057 with serogroup B, were susceptible to penicillin G, neither of which contained the five-mutation pattern. Levofloxacin resistance was observed in two isolates carrying the T91I mutation in the *gyrA* protein.

**Conclusion:**

Our findings suggest the presence of antimicrobial-resistant *N. meningitidis* in Japan, underscoring the necessity for continuous local surveillance. Additional research is crucial for clarifying the ongoing spread of resistance mechanisms and for establishing effective countermeasures to reduce the clinical burden of invasive meningococcal disease.

## Highlights

• We investigated the genetic variability of *Neisseria meningitidis*.
• Antimicrobial resistance to penicillin G and levofloxacin was identified.
• Sequence typing revealed multiple serogroups and resistance-associated mutations.
• *N. meningitidis* resistance patterns match Japanese clones linked to clonal expansion.
• Continuous genetic surveillance is crucial for controlling invasive meningococcal disease.

*Neisseria meningitidis* is a well-known pathogen responsible for invasive meningococcal disease (IMD), which has the potential to cause devastating outbreaks with a high fatality rate [1]. Although antimicrobial resistance in *N. meningitidis* has historically been rare, penicillin- and fluoroquinolone-resistant strains have recently been identified [2,3]. The risk factors for *N. meningitidis* infections typically include humoral immune dysfunction, particularly, in individuals who have undergone splenectomy or those with congenital complement deficiencies [4]. Lately, the increased use of complement C5 inhibitors, such as eculizumab and ravulizumab, is considered to result in a rise in the population vulnerable to IMD [[5], [6], [7]]. The emergence and ongoing dissemination of antimicrobial-resistant *N. meningitidis* have become a global public health concern in this borderless world [8]; however, limited data are available on this threatening pathogen in Japan. In the present study, we aimed to investigate the bacterial and genetic characteristics of *N. meningitidis* clinical isolates detected in our facility.

From 2018 to August 2023, we reviewed microbiological data for *N. meningitidis* recorded in the clinical microbiology laboratory of Okayama University Hospital, Japan. During the study period, bacterial identification was performed using matrix-assisted laser desorption/ionization time-of-flight mass spectrometry (MALDI Biotyper; Bruker Daltonics, Billerica, MA, USA) and minimum inhibitory concentrations (MICs) of antibiotics were determined by the microdilution method using Dry Plate Eiken (Eiken Chemical Co., Ltd, Tokyo, Japan). The MICs were reconfirmed using Etest (BioMérieux Japan Co. Ltd., Tokyo, Japan) as well. We adopted MIC break points provided by the Clinical & Laboratory Standards Institute (M100-33rd Edition). Subsequently, whole genome sequencing was performed using the MiSeq platform (Illumina, San Diego, CA, USA) to identify the genetic determinants contributing to antimicrobial resistance.

Five clinical strains of *N. meningitidis* were isolated in our hospital over a 5-year period (2018-2022); two from eye discharge, two from sputum, and one from cerebrospinal fluid. Although all isolates had been preserved in frozen storage, two failed to regrow. Thus, antibiotic susceptibility testing and genetic analysis were conducted on the remaining three isolates.

The results of antimicrobial susceptibility testing for the clinical isolates are summarized in Table 1. One isolate (isolate 1) demonstrated non-susceptibility to penicillin G, whereas isolate 2 and 3 were susceptible. MICs determined by the Etest method revealed higher values, indicating resistance in isolate 1 and an intermediate in the other two. All isolates were susceptible to cefotaxime, meropenem, azithromycin, and rifampicin. However, isolate 1 and 3 exhibited resistance to levofloxacin.

**Table 1:** Antimicrobial susceptibility of *Neisseria meningitidis* clinical isolates.

	Isolate 1 (Eye discharge)				Isolate 2 (Cerebrospinal fluid)				Isolate 3 (Sputum)			
	MBD		Etest		MBD		Etest		MBD		Etest	
	MIC		MIC		MIC		MIC		MIC		MIC	
Penicillin G	0.25	I	0.38	R	≤0.06	S	0.125	I	≤0.06	S	0.094	I
Ampicillin	0.5	I			≤0.12	S			≤0.12	S		
Ampicillin/sulbactam	0.5	NA			≤0.12	NA			≤0.12	NA		
Cefazolin	2	NA			0.5	NA			0.5	NA		
Cefotaxime	≤0.06	S	0.003	S	≤0.06	S	0.006	S	≤0.06	S	0.004	S
Ceftriaxone	≤0.25	n.d.			≤0.25	n.d.			≤0.25	n.d.		
Cefepime	≤0.25	NA			≤0.25	NA			≤0.25	NA		
Imipenem	0.25	NA			≤0.12	NA			≤0.12	NA		
Meropenem	≤0.06	S	0.047	S	≤0.06	S	0.012	S	≤0.06	S	0.012	S
Erythromycin	0.5	NA			≤0.25	NA			≤0.25	NA		
Clarithromycin	≤0.25	NA			≤0.25	NA			≤0.25	NA		
Azithromycin	1	S	1.5	S	0.25	S	0.75	S	0.5	S	1	S
Clindamycin	>2	NA			>2	NA			>2	NA		
Minocycline	≤1	S			≤1	S			≤1	S		
Levofloxacin	≤1	n.d.	0.25	R	≤1	n.d.	0.012	S	≤1	n.d.	0.25	R
Sulfamethoxazole/Trimethoprim	>40	R			>40	R			≤10	n.d.		
Rifampicin	-	-	0.032	S	-	-	0.006	S	-	-	0.012	S

Interpretations of MIC (μg/ml) were based on the Clinical & Laboratory Standards Institute (M100-33rd Edition).

I, intermediate; MIC, minimum inhibitory concentration; MBD, microbroth dilution; NA, not applicable; n.d., not determinable; R, resistance; S, susceptible.

Multilocus sequence typing using draft genome sequences identified diverse sequence types (STs) and serogroups among the three isolates (Table 2). The genome sequence data supporting the findings are available in the GenBank database at National Center for Biotechnology Information (https://www.ncbi.nlm.nih.gov/). The associated BioProject and BioSample identifiers are PRJNA1179730, SAMN44502379, SAMN44502380, and SAMN44502381, respectively. Isolate 1 belonged to ST-11026 (ST-32 complex) and was non-groupable for serogroup. Isolate 2 was identified as ST-1655 (ST-23 complex) with serogroup Y, whereas isolate 3 belonged to ST-2057 with serogroup B. Isolate 1 exhibited an intermediate to resistant phenotype to penicillin G, which is considered to be associated with the presence of *penA* allele 33. This strain harbored a five-mutation pattern (F504L, A510V, I515V, H541N, and I566V) in the *penA* amino acid sequence, along with additional mutations (XXXIV and N513Y) characteristic of a mosaic *penA* gene. In contrast, isolates 2 and 3 were susceptible to penicillin G and possessed *penA* alleles 22 and 1, respectively, neither of which contained the five-mutation pattern. Levofloxacin resistance was observed in isolates 1 and 3, both of which carried the T91I mutation in the *gyrA* protein (alleles 376 and 348, respectively). Isolates 2 remained susceptible to levofloxacin, with *gyrA* allele 1.

**Table 2:** Results of whole genome sequencing of *Neisseria meningitidis* clinical isolates.

Strain	Sequence type	Serogroup	*penA* allele	Five mutations in *penA*^a^	NG-STAR PCG	*gyrA* allele
Isolate 1 (Eye discharge)	11026	Non-groupable	33	+	XXXIV, N513Y mosaic	376:T91I
Isolate 2 (Cerebrospinal fluid)	1655	Y	22	−	−	1
Isolate 3 (Sputum)	2057	B	1	−	−	348:T91I

a Five mutations: F504L, A510V, I515V, H541N, I566V in PenA amino-acids sequence.

Despite the limited number of strains analyzed and the absence of preceding oversee traveling history and previous vaccinations of the patients, our findings underscore the increasing prevalence of penicillin- and fluoroquinolone-resistant *N. meningitidis* clinical strains in Japan [3,8]. Notably, the identification of *penA* mosaic alleles and *gyrA* T91I mutations in our isolates matches resistance patterns observed in specific Japanese clones, such as ST-11026 and cc2057, which are believed to have spread through clonal expansion [3]. The genetic variability observed among these isolates suggests that antimicrobial resistance in *N. meningitidis* may have been driven by clonal expansion and horizontal gene transfer, as seen in other *Neisseria* species through homologous recombination [2,9]. This underlines the importance of continuous genetic surveillance to detect the emergence of novel resistant strains.

The isolation of fluoroquinolone-resistant strains in our facility suggests the need for caution when considering prophylactic or therapeutic agents. Notably, increasing prevalence of fluoroquinolone-resistant *N. meningitidis* strains has been documented across multiple regions, including North America [2,10], Europe [11,12], and Japan [3,13]. Regular revision of regional antimicrobial treatment guidelines, informed by current resistance surveillance data, is essential for optimizing therapeutic outcomes, despite limited availability of comprehensive resistance pattern databases.

In summary, although the limited sample size of three meningococcal isolates constrains the generalizability of these findings, this case series, complemented by genomic analysis, demonstrates the emergence of antimicrobial-resistant *N. meningitidis* strains in Japan, emphasizing the importance of regional surveillance systems. Further investigations are essential to deepen our understanding of resistance mechanisms in *N. meningitidis* and to develop effective strategies to mitigate the clinical impact of IMD.

## Funding

This research did not receive any specific grant from funding agencies in the public, commercial, or not-for-profit sectors.

## Ethical approval statement

A need of obtaining informed consent was waived because the study does not include identifiable information of patients.

## Author contributions

HH conceived the study; ST, KG, and KI performed the microbiological testing; KG and SF drafted the manuscript; HH revised the manuscript; OM and HH supervised the study. All authors interpreted the results and gave final approval to the submitted manuscript.

## Availability of data and materials

The data sets used during the current study are available from the corresponding author upon reasonable request.

## Declarations of competing interest

The authors have no competing interests to declare.

## References

[1] Morello BR, Milazzo A, Marshall HS, Giles LC. (2024). Public health management of invasive meningococcal disease outbreaks: worldwide 1973–2018, a systematic review. BMC Public Health.

[2] Potts CC, Retchless AC, McNamara LA, Marasini D, Reese N, Swint S (2021). Acquisition of ciprofloxacin Resistance among an Expanding Clade of β-lactamase-Positive, serogroup Y Neisseria meningitidis in the United States. Clin Infect Dis.

[3] Saito R, Nakajima J, Prah I, Morita M, Mahazu S, Ota Y (2022). Penicillin- and ciprofloxacin-resistant invasive Neisseria meningitidis isolates from Japan. Microbiol Spectr.

[4] Lewis LA, Ram S. (2020). Complement interactions with the pathogenic Neisseriae: clinical features, deficiency states, and evasion mechanisms. FEBS Lett.

[5] Yu Z-Y, Tsai M-J, Lin Y-J, Liu W-D, Chou S-C, Hung C-C. (2020). Disseminated gonococcal infection in a patient with paroxysmal nocturnal hemoglobinuria having received ravulizumab and meningococcal vaccine. J Microbiol Immunol Infect.

[6] Kang C. (2023). Ravulizumab: A review in generalised myasthenia gravis. Drugs.

[7] Galli N, Pettine L, Panigada M, Daprai L, Suriano G, Grancini A (2023). Non-capsulated Neisseria meningitidis sepsis in a paroxysmal nocturnal hemoglobinuria patient treated with ravulizumab: case report and review of the literature. Front Immunol.

[8] Takahashi H, Morita M, Kamiya H, Fukusumi M, Yasuda M, Sunagawa M (2023). Emergence of ciprofloxacin- and penicillin-resistant Neisseria meningitidis isolates in Japan between 2003 and 2020 and its genetic features. Antimicrob Agents Chemother.

[9] Hong E, Deghmane A-E, Taha M-K. (2018). Acquisition of beta-lactamase by Neisseria meningitidis through possible horizontal gene transfer. Antimicrob Agents Chemother.

[10] Tsang RSW, Law DKS, Deng S, Hoang L. (2017). Ciprofloxacin-resistant Neisseria meningitidis in Canada: likely imported strains. Can J Microbiol.

[11] Vacca P, Vocale C, Fazio C, Ambrosio L, Luzi K, Neri A (2019). Invasive meningococcal disease due to ciprofloxacin-resistant Neisseria meningitidis Sequence Type 7926: the first case in Italy, likely imported. J Glob Antimicrob Resist.

[12] Tzanakaki G, Georgakopoulou T, Xirogianni A, Papandreou A, Deghmane A-E, Magaziotou I (2020). First report of meningococcal ciprofloxacin resistance in Greece due to invasive isolates of the sequence type ST-3129. Eur J Clin Microbiol Infect Dis.

[13] Kawasaki Y, Matsubara K, Takahashi H, Morita M, Ohnishi M, Hori M (2018). Invasive meningococcal disease due to ciprofloxacin-resistant Neisseria meningitidis sequence type 4821: the first case in Japan. J Infect Chemother.

